# CX-4945 Induces Methuosis in Cholangiocarcinoma Cell Lines by a CK2-Independent Mechanism

**DOI:** 10.3390/cancers10090283

**Published:** 2018-08-23

**Authors:** Jomnarong Lertsuwan, Kornkamon Lertsuwan, Anyaporn Sawasdichai, Nathapol Tasnawijitwong, Ka Ying Lee, Philip Kitchen, Simon Afford, Kevin Gaston, Padma-Sheela Jayaraman, Jutamaad Satayavivad

**Affiliations:** 1 Laboratory of Chemical Carcinogenesis, Chulabhorn Research Institute, Bangkok 10210, Thailand; jomnarong@cri.or.th (J.L.); anyaporn@cri.or.th (A.S.); 2 Department of Biochemistry, Faculty of Science, Mahidol University, Rama VI Road, Bangkok 10400, Thailand; kornkamon.ler@mahidol.edu; 3 Center of Calcium and Bone Research (COCAB), Faculty of Science, Mahidol University, Rama VI Road, Bangkok 10400, Thailand; 4 Laboratory of Pharmacology, Chulabhorn Research Institute, Bangkok 10210, Thailand; nathapol@cri.or.th; 5 Institute of Cancer and Genomic Sciences, University of Birmingham, Birmingham B15 2TT, UK; kxl647@student.bham.ac.uk (K.Y.L.); p.kitchen@bham.ac.uk (P.K.); 6 Institute of Immunology and Immunotherapy, University of Birmingham, Birmingham B15 2TT, UK; s.c.afford@bham.ac.uk; 7 Division of Cancer and Stem Cells, School of Medicine, University of Nottingham, Nottingham NG7 2RD, UK; kevin.gaston@nottingham.ac.uk

**Keywords:** protein kinase CK2, CX-4945, cholangiocarcinoma, non-canonical cell death, methuosis

## Abstract

Cholangiocarcinoma is a disease with a poor prognosis and increasing incidence and hence there is a pressing unmet clinical need for new adjuvant treatments. Protein kinase CK2 (previously casein kinase II) is a ubiquitously expressed protein kinase that is up-regulated in multiple cancer cell types. The inhibition of CK2 activity using CX-4945 (Silmitasertib) has been proposed as a novel treatment in multiple disease settings including cholangiocarcinoma. Here, we show that CX-4945 inhibited the proliferation of cholangiocarcinoma cell lines in vitro. Moreover, CX-4945 treatment induced the formation of cytosolic vacuoles in cholangiocarcinoma cell lines and other cancer cell lines. The vacuoles contained extracellular fluid and had neutral pH, features characteristic of methuosis. In contrast, simultaneous knockdown of both the α and α′ catalytic subunits of protein kinase CK2 using small interfering RNA (siRNA) had little or no effect on the proliferation of cholangiocarcinoma cell lines and failed to induce the vacuole formation. Surprisingly, low doses of CX-4945 increased the invasive properties of cholangiocarcinoma cells due to an upregulation of matrix metallopeptidase 7 (MMP-7), while the knockdown of CK2 inhibited cell invasion. Our data suggest that CX-4945 inhibits cell proliferation and induces cell death via CK2-independent pathways. Moreover, the increase in cell invasion brought about by CX-4945 treatment suggests that this drug might increase tumor invasion in clinical settings.

## 1. Introduction

Cholangiocarcinoma (CCA) is a cancer of bile duct epithelia thought to arise as a result of chronic inflammation of the liver and bile ducts. The incidence of CCA has increased gradually worldwide during the last three decades [1,2,3,4]. The incidence of this disease is highest in Southeast Asia [5] particularly in the northeastern provinces of Thailand due to persistent liver fluke infection [6,7]. Although, surgery is considered the best treatment option, most patients are not suitable for surgery on presentation [8,9]. Moreover, approximately 70% of intrahepatic cholangiocarcinoma patients have a recurrence of disease post-surgery [10]. Adjuvant capecitabine treatment is effective post-surgery but median survival is only around four years [11]. Non-resectable disease can be treated using radiation and gemcitabine and cisplatin as first-line chemotherapy. However, median overall survival remains very poor at only around 12 months [12]. Therefore, there is an urgent unmet clinical need for new adjuvant therapies that could prevent recurrence as well as for new and effective second line drug treatments.

Protein kinase CK2 (previously casein kinase II), a ubiquitously expressed serine/threonine kinase, forms a tetrameric holoenzyme composed of two catalytic subunits, which are alpha (α) or alpha′ (α′), or a combination of both, and two regulatory beta (β) subunits [13]. CK2 levels in many cancer types are abnormally high [14]. CK2 promotes cell survival and was shown to suppress apoptosis in various cancer types, such as HeLa and the gastric cancer cell line SGC-7901, where CK2 phosphorylated apoptosis repressor with caspase recruitment domain (ARC) [15]. In addition, CK2 promoted malignant peripheral nerve sheath tumors (MPNST) cell survival by stabilizing β-catenin [16]. Downregulation of CK2 with short hairpin RNA (shRNA) or with CX-4945 also known as Silmitasertib, a CK2 inhibitor, caused MPNST cells to arrest in the G2/M phase and undergo apoptosis [16]. Downregulation of CK2 with small interfering (siRNA) or by using CX-4945 induced apoptosis in non-small cell lung cancer cell lines by downregulation of Akt and its downstream signals [17]. Similarly, apoptosis was observed in acute myeloid leukemia cells by a p53-dependent mechanism after CK2 knockdown or CX-4945 application [18]. Furthermore, stable knockdown of CK2 sensitized HEp-2 laryngeal carcinoma cells to cisplatin-induced apoptosis [19].

CK2 has been proposed as a drug target for many cancers. Downregulation of protein kinase CK2 induced the senescent phenotype in human diploid fibroblast IMR-90 cells [20], and decreased androgen receptor nuclear translocation, which led to a reduced expression of genes required for prostate cancer cell proliferation [21]. CX-4945 has also been shown to decrease the survival of many cancer cell types including bladder [22], hematological [23,24], prostate [25], and breast cancer cells [26]. CX-4945 up to 10 µM showed an inhibition on Akt phosphorylation in prostate cancer, PC3 cells, and [27] non-small cell lung cancer cells, H1299, Calu-1, and H358 [17]. It also induced cell cycle arrest and apoptosis in the aforementioned prostate and non-small cell lung cancer cell lines [17,27]. An in vivo study showed that CX-4045 up to 75 mg/kg was safe in mice and inhibited PC3 tumor xenograft growth [27]. In primary human mucin- and mixed-intrahepatic CCA, CX-4945 up to 50 µM significantly decreased the viability of primary human cell cultures and induced apoptosis at 10 µM, whereas Cytokeratin 19 (CK19)-positive cells required a higher dose at 30 µM [28]. CX-4945 increased the number of γ-H2AX positive cells that are a marker for DNA double-strand breaks [28]. However, inhibition of DNA-PK (DNA-dependent protein kinase), previously reported as downstream of CK2 in glioblastoma cells [29], unexpectedly reduced γ-H2AX positive cells in primary human intrahepatic CCA cells [28]. This suggested that DNA-PK and also possibly CK2 in the setting of CCA acted as negative modulators of DNA double strand break repair, which is opposite from the mechanism reported in glioblastoma [29] and leukemia [30].

Interestingly, CX-4945 at 1 and 10 µM was recently shown to have off-target effects. The inhibition of Cdc2-like kinases (Clks) from these doses of CX-4945 resulted in a suppression of phosphorylation of serine/arginine (S/R) rich proteins in mammalian cells [31]. In addition, CX-4945 has been shown to affect alternative splicing of a wide range of genes, including CK2 itself, in a CK2-independent manner in the HEK293T cell line [31].

Although apoptosis is the most widely recognized form of physiological programmed cell death, several non-apoptotic cell death mechanisms have been reported to date. One distinct characteristic of many non-apoptotic cell death mechanisms is the formation of cytoplasmic vacuoles. The origin of the cytoplasmic vacuoles in different non-canonical cell death mechanisms is different yet not unique. One relatively well known non-apoptotic cell death is autophagy. Autophagy forms autophagosomes that are double-membrane vacuoles [32,33,34]. In contrast, methuosis, a recently described cell death mechanism, utilizes single-membrane vacuoles from macropinocytosis [35,36,37]. A few other types of non-apoptotic cell death mechanisms share a similar origin for the formation of cytoplasmic vacuoles. Oncosis, paraptosis, and necroptosis form vacuoles from endoplasmic reticulum (ER) and mitochondria [35,38,39,40,41,42].

Methuosis is derived from the Greek methuo (to drink to intoxication) because the hallmark of this form of cell death is the formation of large fluid-filled vacuoles derived from macropinosomes. The demise of the cell resembles many forms of necrosis, with loss of metabolic capacity and plasma membrane integrity. Moreover, cell shrinkage and nuclear fragmentation associated with apoptosis are not observed [35]. Methuosis was initially defined in glioblastoma cells after ectopic expression of activated Ras [36,37], but recent reports have described small molecules, including indole-based chalcone 3-(2-methyl-1H indol-3-yl)-1-(4-pyridinyl)-2-propen-1-one (MIPP) and MOMIPP, which has a 5-methoxy group added to the indole ring of MIPP, that can induce the features of methuosis [37,43].

Herein, we report for the first time that CX-4945 at low doses stimulated CCA cell invasion due to an upregulation of matrix metallopeptidase 7 (MMP-7) and matrix metallopeptidase 9 (MMP-9). Also, CX-4945 induced cytosolic vacuolization, which met the criteria of methuosis, via a CK2-independent mechanism in a dose- and time-dependent manner.

## 2. Results

### 2.1. CX-4945 Inhibits CK2 Activity at High Dose

**Figure 1:**
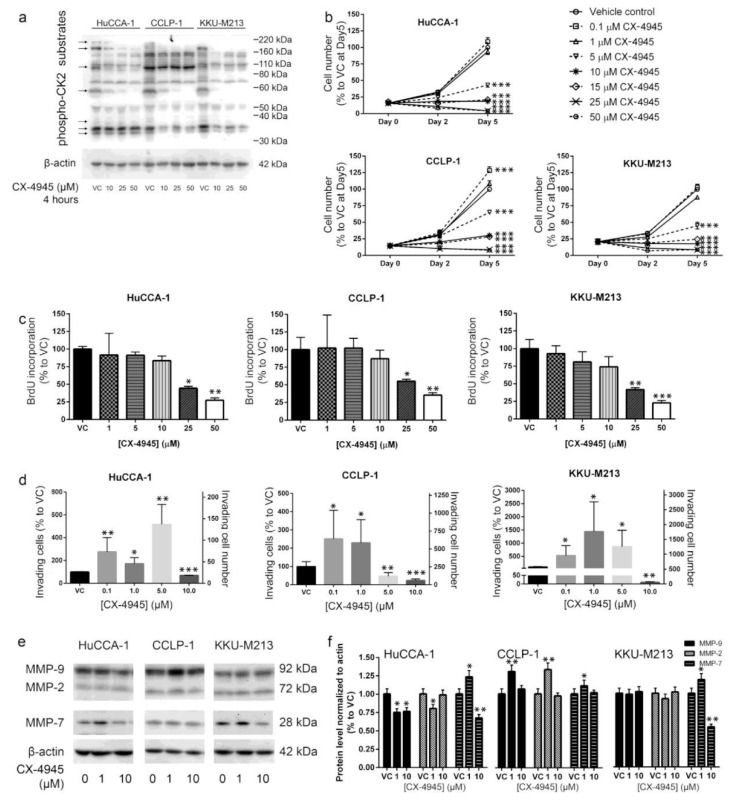
The effects of CX-4945 on cholangiocarcinoma (CCA) cell lines. (**a**) CX-4945 (Silmitasertib) inhibited CK2 (previously casein kinase II) activity in a dose-dependent manner. Arrows indicate CK2 phospho-substrates that decrease in intensity with CX-4945 treatment. (**b**) CX-4945 at 5 µM and higher doses reduced CCA cell number in three CCA cell lines as determined using MTT assays. (**c**) CX-4945 at 25 and 50 µM inhibited CCA cell lines proliferation in a 5-bromo-2′-deoxyuridine (BrdU) incorporation assay 24 h post-treatment. (**d**) CX-4945 exerted a biphasic effect on cell invasion through Matrigel. CX-4945 at 5 µM or lower doses induced invasion by HuCCA-1 cells and KKU-M213 cells while CX-4945 at 10 µM inhibited CCA cell invasion through Matrigel. (**e**) Twenty-four hours’ post-treatment of CX-4945 at low doses upregulated matrix metallopeptidase 7 (MMP-7) level while 10 µM CX-4945 downregulated MMP-7 level. MMP-2 and MMP-9 levels were not altered. (**f**) Quantitative data of protein level changes post treatment of CX-4945 in panel (**e**). VC; vehicle control, *; *p* < 0.05, **; *p* < 0.01, ***; *p* < 0.001. All experiments were performed in triplicate and with at least at three biological replicates. Graphs were plotted as mean ± SEM.

To investigate the effects of CX-4945 on CCA cells, we treated three CCA cell lines with increasing amounts of this drug. CX-4945 reduced CK2 activity in a dose-dependent manner 4 h post-treatment as determined using Western blotting with an antibody that recognizes phosphorylated CK2 sites in multiple proteins (Figure 1a). The CCA cell lines all showed a reduction in multiple phospho-CK2 substrates with apparent molecular masses of approximately 200, 180, 100, 60, 37, 35, and 33 kDa as indicated by arrows in the Figure (Figure 1a).

### 2.2. CX-4945 Treatment Inhibits CCA Cell Proliferation

To determine the effects of CX-4945 on CCA cell proliferation we treated the three cell lines described above and examined the effect on cell number and 5-bromo-2′-deoxyuridine (BrdU) incorporation. After 5 days of treatment, CX-4945 at 5 µM or higher doses reduced CCA cell number in all of the cell lines (Figure 1b). CX-4945 at 5 µM reduced CCA cell number to approximately 50% of the vehicle control in HuCCA-1, KKU-M213 cells and in CCLP-1 approximately to 70% as compared to vehicle control group at 5-days post-treatment. CCA cells treated with 10 and 15 µM CX-4945 did not increase in number over 5 days in culture (Figure 1b), and higher doses of CX-4945 (25 and 50 µM) decreased cell number significantly at 5 days after treatment (Figure 1b). To determine whether the reduction in cell number is accompanied by reduced cell proliferation, we examined the effects of CX-4945 on 5-bromo-2′-deoxyuridine (BrdU) incorporation. CX-4945 at 25 and 50 µM inhibited BrdU incorporation on all CCA cell lines by approximately 50% and 25%, respectively, at 24 h post-treatment (Figure 1c). A slightly lower inhibition was observed on CCLP-1 cells (Figure 1c).

### 2.3. CX-4945 Treatment Alters Cell Invasion

Protein kinase CK2 is known to be important in cell migration and cancer cell invasion. To determine the effects of CX-4945 on CCA cell invasion we examined the ability of the cells to traverse a layer of Matrigel in vitro. CX-4945 treatment showed biphasic effects on CCA cell invasion though Matrigel. CX-4945 at 10 µM significantly inhibited cell invasion through Matrigel in the three CCA cell lines tested (Figure 1d). In contrast, lower concentrations of CX-4945 stimulated invasion in all CCA cell lines tested (Figure 1d). The increase in cell invasion at low CX-4945 doses was not due to an increase in cell number as the assays were performed at the same time point (24 h post-treatment) that was shown by BrdU assay to have equivalent proliferation rates between the control and CX-4945 treated groups (1 and 5 µM) (Figure 1c). In addition, MTT assay at a later time point (48 h post-treatment) also showed no difference in cell number between these groups (Figure 1b). The increase in cell invasion was at least in part due to an increase in MMP-9, MMP-7, and matrix metallopeptidase 2 (MMP-2) levels in CCLP-1, and an increase in MMP-7 levels in HuCCA-1 and KKU-M213 (Figure 1e,f). The decrease in cell invasion at 10 µM of CX-4945 was at least in part due to a decrease in MMP-9 and MMP-7 levels in HuCCA-1 and to MMP-7 levels in KKU-M213. In addition to a decrease in MMP levels, a smaller invasion in the 10 µM CX-4945-treated group was also likely to be a consequence of the inhibition of cell proliferation at this dose (Figure 1b,c). We conclude that at lower doses, CX-4945 treatment increased the ability of CCA cells to invade Matrigel, while higher doses inhibited this ability.

### 2.4. CX-4945 Treatment Induces Intensive Vacuolization

**Figure 2:**
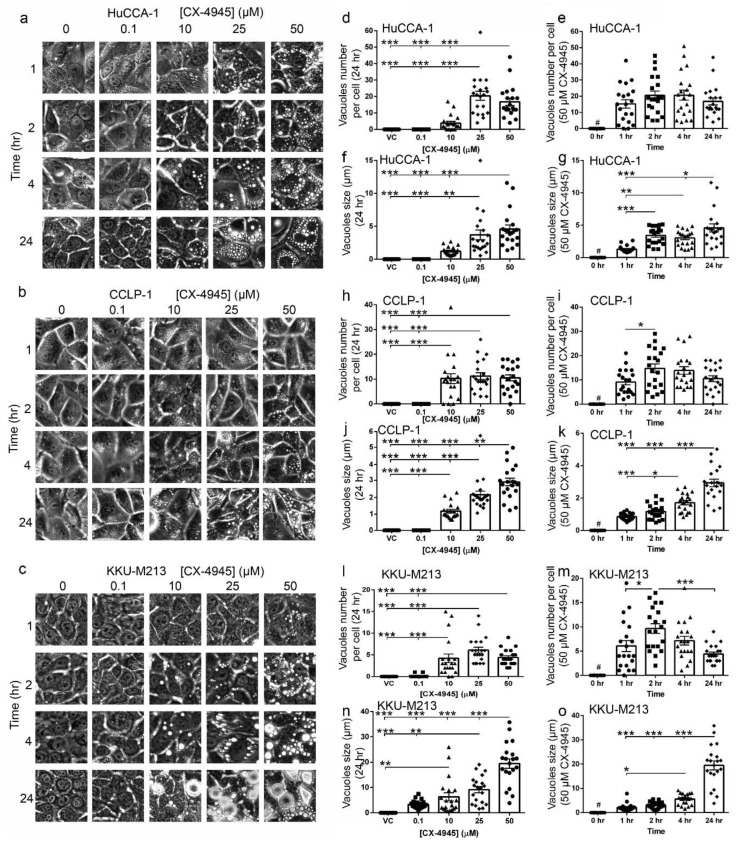
CX-4945 induced vacuolization. (**a**–**c**) CX-4945 induced intensive vacuolization in CCA cells. CX-4945 induced vacuolization in CCA cell lines in a dose- (**d**,**f**,**h**,**j**,**l**,**n**) and time- (**e**,**g**,**i**,**k**,**m**,**o**) dependent manner. VC; vehicle control, *; *p* < 0.05, **; *p* < 0.01, ***; *p* < 0.001, #; significantly different from all other groups at *p* < 0.001. All experiments were performed with at least three biological replicates. Graphs were plotted as mean ± SEM.

**Figure 3:**
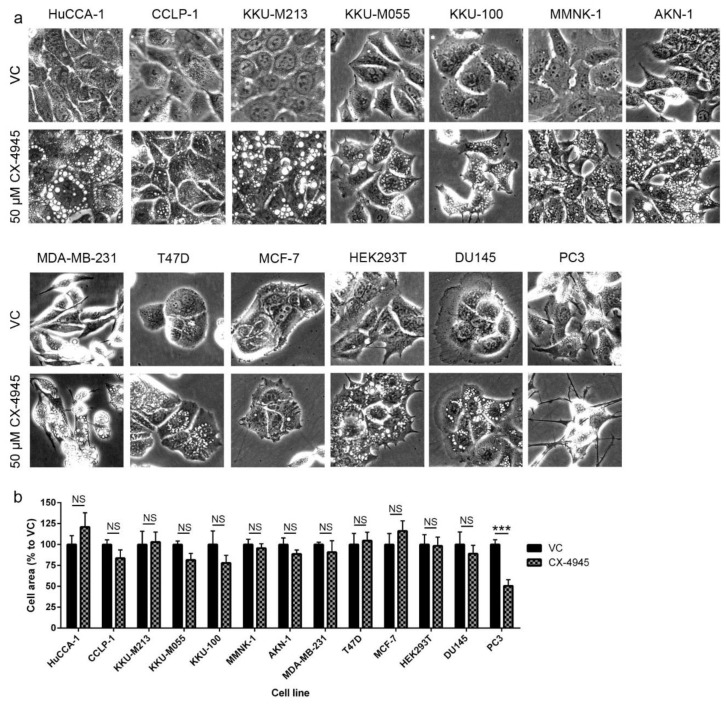
CX-4945 induced vacuolization in a wide range of cancer and immortalized cell lines. (**a**) CCA cells (HuCCA-1, CCLP-1, KKU-M213, KKU-M055, and KKU-100) and immortalized cholangiocytes (MMNK-1 and AKN-1), breast cancer cells (MDA-MB-231 and T48D), prostate cancer cells (DU145), and human embryonic kidney cells (HEK293T) form intensive cytoplasmic vacuoles at 4 h after CX-4945 treatment. Breast cancer cells, MCF-7, formed only few vacuoles in small number of cells, while prostate cancer cell, PC3, did not form any vacuoles. (**b**) Cell area of cell lines that formed vacuoles did not altered, but PC3 cell area decreased to approximately 50% as compared to the vehicle control group. VC; vehicle control, ***; *p* < 0.001, NS; not significant. Pictures are representative of the results from three independent experiments. Graphs were plotted as mean ± SEM.

Prominent vacuoles were observed as soon as 1 h after CX-4945 treatment in all CCA cell lines tested (Figure 2a–c). The number of the vacuoles at 24 h post-treatment increased in a dose-dependent manner in CX-4945 treated HuCCA-1, CCLP-1, and KKU-M213 cells (Figure 2d,h,l). The number of vacuoles also increased in a time-dependent manner until 4 h post-treatment in HuCCA-1 (Figure 2e) or 2 h after treatment in CCLP-1 and KKU-M213 (Figure 2i,m) before declining at later time points. This reduction in vacuole number might be due to the fusion of small vacuoles into larger ones. In keeping with this view, the size of the vacuoles increased in a dose- and time-dependent manner (Figure 2f,g,j,k,n,o). The vacuoles filled most of the cytoplasm at 24 h after treatment in cells treated with 50 µM CX-4945 (Figure 2a–c). To determine whether vacuole formation is limited to CCA cells, we next treated a variety of other CCA cells and non-CAA cells with CX-4945. CX-4945 treatment induced vacuole formation in all of the additional CCA cell lines tested (Figure 3a). Furthermore, 50 µM CX-4945 treatment at 4 h also induced intensive vacuolization in immortalized cholangiocytes (MMNK-1 and AKN-1 cells), as well as breast cancer cell lines (MDA-MB-231 and T47D), prostate cancer cell line DU145, and human embryonic kidney cells (HEK293T). However, one prostate cancer cell line (PC3) did not form vacuoles at all under the conditions tested, and one breast cancer cell line (MCF-7) formed only a few vacuoles in some cells (Figure 3a). Interestingly, although the cell area of the cell lines that form vacuoles did not decrease (Figure 3b), PC3 cell area decreased approximately 50% as compared to the vehicle control group (Figure 3b).

### 2.5. CX-4945 Induces Caspase-3 Independent Non-Autophagic Cell Death in CCA Cells

**Figure 4:**
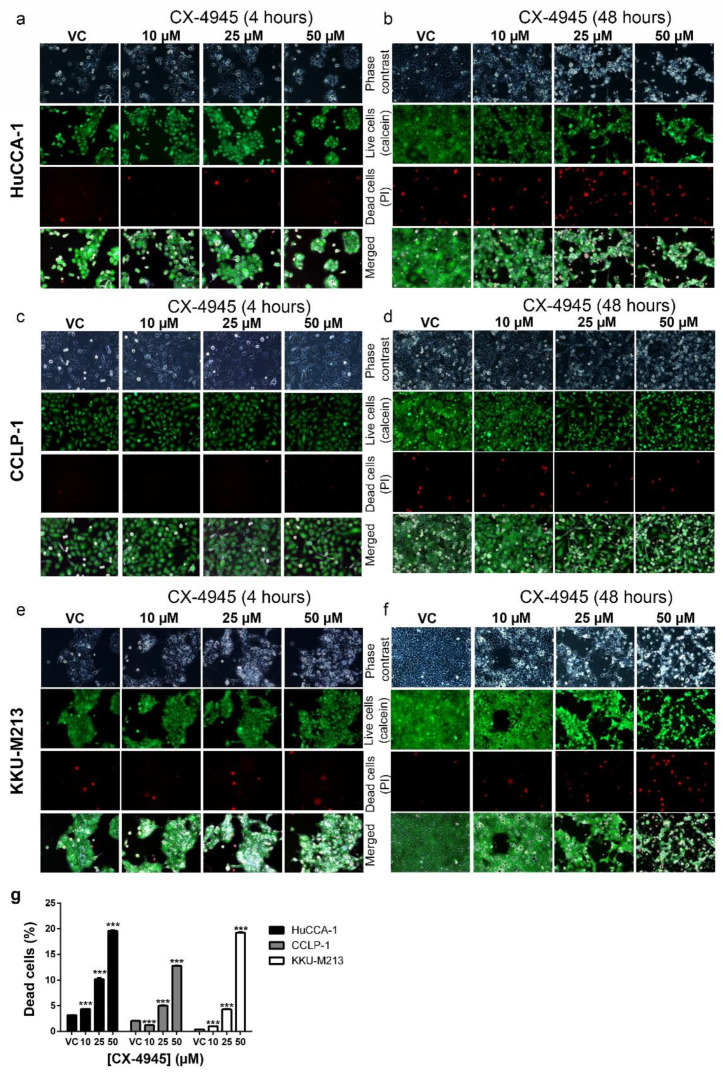
CX-4945 induced cell death. Propidium iodide staining showed that treatment with CX-4945 for 4 h did not induce cell death in (**a**) HuCCA-1, (**c**) CCLP-1, and (**e**) KKU-M213 cells. Extended CX-4945 treatment at 48 h, however, induced extensive cell death in (**b**) HuCCA-1 and (**f**) KKU-M213 cell lines. (**d**) Dead cell number was not increased in CCLP-1 treated with CX-4945 but live cell number clearly decreased in the 25 and 50 µM CX-4945 treated samples as well as in (**b**) HuCCA-1 and (**f**) KKU-M213 cell lines. (**g**) Percentage of dead cell increased in a dose-dependent manner in all three CCA cell lines. ***; *p* < 0.001, Pictures are representative of the results from three independent experiments.

**Figure 5:**
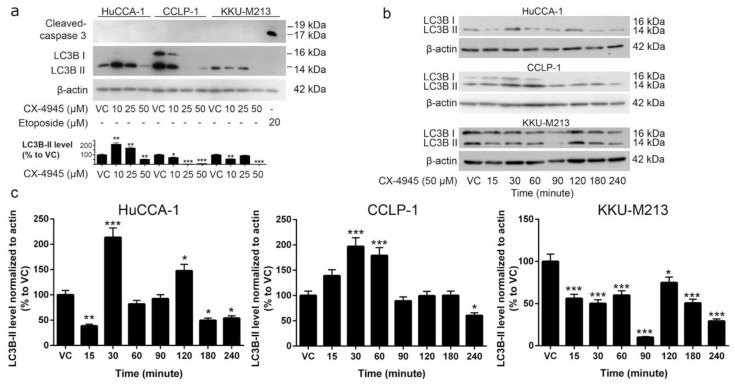
CX-4945 induced non-apoptotic, non-autophagic cell death. (**a**) The apoptotic marker, cleaved caspase-3, and autophagic marker, LC3B-II, was not increased after 48 h of CX-4945 treatment when dead cells and vacuoles were observed. (**b**,**c**) The level of LC3B-II in HuCCA-1 and CCLP-1 cells increased at early time points, and then decreased after 3 h of 50 µM CX-4945 treatment in HuCCA-1 cells and after 4 h in CCLP-1 cells. The level of LC3B-II in KKU-M213 decreased as soon as 15 min after 50 µM CX-4945 treatment. VC; vehicle control, *; *p* < 0.05, **; *p* < 0.01, ***; *p* < 0.001. All experiments were performed with at least three replicates. Graphs were plotted as mean ± SEM.

To determine whether vacuolization was accompanied by increased cell death, we treated CCA cells with CX-4945 and stained live cells with calcein and dead cells with propidium iodide. No significant increase in the number of dead cells was observed after 4 h of CX-4945 treatment (Figure 4a,c,e), which was the time point where a decreased level of CK2 activity was observed with CX-4945 treatment (Figure 1a). However, CX-4945 at 25 and 50 µM significantly induced cell death in HuCCA-1 cells (Figure 4b) and KKU-M213 cells (Figure 4f) at 48 h post-treatment. Although there was no significant increase in the number of dead CCLP-1 cells following treatment with CX-4945, the total number of cells 48 h post-treatment was clearly reduced (Figure 4d, calcein panels). This was also seen in the other cell lines (Figure 4b,f). The percentage of dead cells was increased in a dose-dependent manner from approximately 1–3% in the vehicle control group to 12% (CCLP-1) or 20% (HuCCA-1 and KKU-M213) in 50 µM treated group (Figure 4g). Interestingly, the number of apoptotic cells did not increase in these cells after treatment with CX-4945 at any concentration for 48 h (Figure 5a), while cell death was detected at 48 h post-treatment. Etoposide treated KKU-M213 cells were used as positive control for the cleaved caspase-3 apoptosis assay. Furthermore, the vacuoles that presented after CX-4945 treatment do not appear to be autophagosomes since levels of the autophagosomal marker LC3B-II decreased (Figure 5a) while the number and size of the vacuoles increased (Figure 2). Although HuCCA-1 treated with 10 and 25 µM CX-4945 showed an increased level of LC3B-II, the cells treated with 50 µM, which is the concentration where the highest number of vacuoles was observed, showed a decreased level of LC3B-II. Moreover, a decreased level of LC3B-II was observed in both CCLP-1 and KKU-M213 treated with CX-4945 (Figure 5a). To further examine the involvement of LC3B-II in the vacuolization induced by CX-4945, a time-course treatment of 50 µM CX-4945 was performed. The level of LC3B-II was elevated at early time points post-treatment in HuCCA-1 and CCLP-1 cells, but it decreased significantly at 4 h post-treatment. A similar trend was observed in KKU-M213 cells where the level of LC3B-II at earlier time points was higher than that at 4 h (Figure 5b,c). We conclude that CX-4945 is likely to induce non-canonical cell death in these cell lines. However, the LC3B-II protein might be involved in the initiation of vacuole formation.

### 2.6. CX-4945 Induced Methuosis in KKU-M213

**Figure 6:**
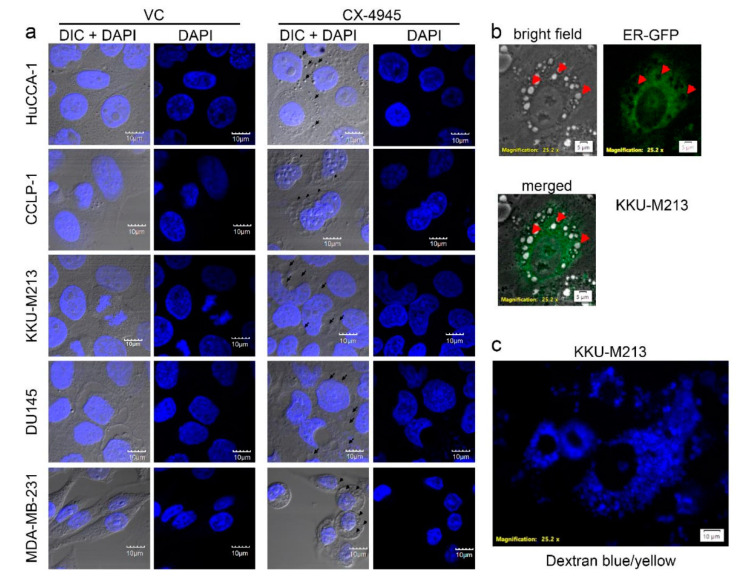
CX-4945 induced methuosis in CCA cell line, KKU-M213. (**a**) CX-4945 induced cytoplasmic vacuolization in three CCA cell lines (HuCCA-1, CCLP-1, and KKU-M213) prostate cancer cell line (DU145), and breast cancer cell line (MDA-MB-231), while it left the nucleus intact as compared to the vehicle control group. (**b**) The endoplasmic reticulum (ER) of KKU-M213 showed no sign of swelling during vacuolization induced by CX-4945. (**c**) The vacuoles induced by CX-4945 contained dextran blue/yellow, which was added in the culture media during CX-4945 incubation. The presence of dextran blue/yellow in the vacuoles indicated that cell engulfed extracellular fluid and formed vacuoles. VC; vehicle control, arrow head indicates cytoplasmic vacuole.

All cell lines used in these studies, except PC3 cells which did not form vacuoles (Figure 3a), showed no change in cell area (Figure 3b) and had no membrane blebbing (Figure 3a). Although their cytoplasm was filled with vacuoles, the cell membrane was well-spread and cell shape was normal as compared to the vehicle control group (Figure 3a). To further identify the cell death mechanism induced by CX-4945 in CCA cell lines, the nucleus and ER of cells treated with CX-4945 were examined. First, nuclear staining with DAPI showed that the nucleus of cells treated with 25 µM CX-4945 for 24 h were still intact. No chromatin condensation or nuclear fragmentation was observed (Figure 6a). Second, KKU-M213, which are cells that are most sensitive to CX-4945 in terms of vacuole formation, were incubated for 72 h with CellLight^®^ ER-GFP BacMam 2.0 (C10590, Fisher Scientific, Hampton, NH, USA). This reagent transfected and expressed GFP-fused ER signal sequence, calreticulin, and KDEL, in live cells, thereby labeled ER with GFP. The ER of KKU-M213 treated with CX-4945 covered a large part of cytoplasm but did not overlap with the vacuoles (Figure 6b). Some large vacuoles reside near the nucleus and, therefore, share a similar space as the ER in the cytoplasm, however, many other medium and small vacuoles as well as some other large vacuoles were observed in the area not overlapping with the ER (Figure 6b). In addition, dextran blue/yellow was added in the culture media during CX-4945 treatment to track the endocytosis process. KKU-M213 engulfed dextran blue/yellow into the vacuoles, which showed as blue dots of various sizes in the cytoplasm (Figure 6c). Dextran blue/yellow remained blue indicating that pH of the vacuoles was neutral since it changes to green or yellow when the environment is acidic. We conclude that the vacuoles formed from CX-4945 treatment did not alter nuclear and membrane integrity. Furthermore, vacuoles in KKU-M213 are not formed from the ER, instead they are formed from the endocytosis process as their pH is neutral. These finding meet the criteria for methuosis.

### 2.7. CX-4945 Induced Vacuolization Is CK2-Independent

**Figure 7:**
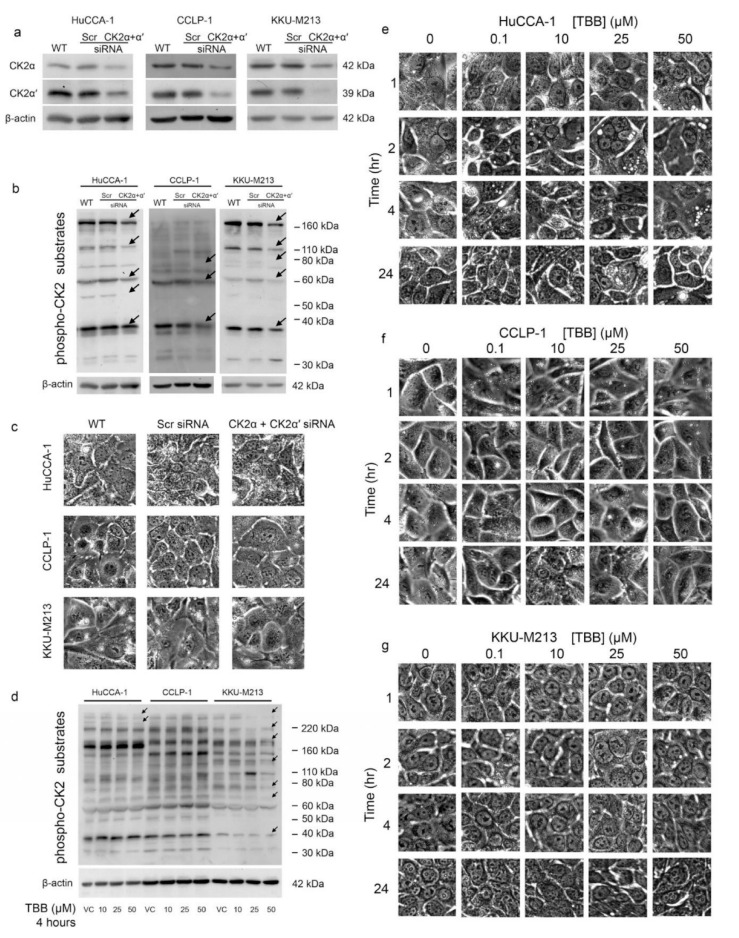
CK2 knockdown and inhibition of CK2 with 4,5,6,7-Tetrabromobenzotriazole (TBB) failed to induce vacuolization. (**a**) Knockdown of α and α′ catalytic subunits of protein kinase CK2 using small interfering (siRNA). (**b**) Knockdown of α and α′ catalytic subunits of protein kinase CK2 decreased phospho-CK2 substrate levels in HuCCA-1, CCLP-1, and KKU-M213 cell lines. (**c**) Knockdown of α and α′ catalytic subunits of protein kinase CK2 failed to induce cytoplasmic vacuolization. (**d**) TBB reduced levels of phospho-CK2 substrate levels in HuCCA-1 and KKU-M213 cell lines. (**e**–**g**) TBB failed to induce cytoplasmic vacuolization. WT; wild type, Scr; scramble siRNA. Pictures are representative of the results from three independent experiments.

To determine whether the effects of CX-4945 on vacuole formation were mediated by the inhibition of CK2, we next knocked down the expression of the α and α′ CK2 catalytic subunits using specific siRNAs (Figure 7a). Knockdown of both CK2 α and α′ in the HuCCA-1, CCLP-1, and KKU-M213 CCA cell lines was verified using Western blotting (Figure 6a). In all three cell lines, knockdown of CK2 α and CK2 α′ decreased the levels of multiple phospho-CK2 substrates (Figure 7b). A reduction in phospho-CK2 substrate levels was observed for proteins with apparent molecular masses of 180, 110, 60, and 37 kDa as indicated by arrows in the Figure (Figure 7b). However, CK2 α and CK2 α′ knockdown failed to induce vacuolization in any of the CCA cell lines (Figure 7c). To confirm these results, we next treated CCA cell lines with 4,5,6,7-Tetrabromobenzotriazole (TBB), a well-characterized inhibitor of CK2 activity. Treatment with TBB did not induce the formation of vacuoles in any of the three CCA cells lines (Figure 7e–g) although it did block the phosphorylation of multiple CK2 substrates in HuCCA-1 and KKU-M213 cell lines (Figure 7d). We conclude that the induction of vacuoles by CX-4945, at least in HuCCA-1 and KKU-M213 cells, is not mediated by the inhibition of CK2 activity.

### 2.8. CK2 Knockdown Inhibited CCA Cell Proliferation and Cell Invasion

**Figure 8:**
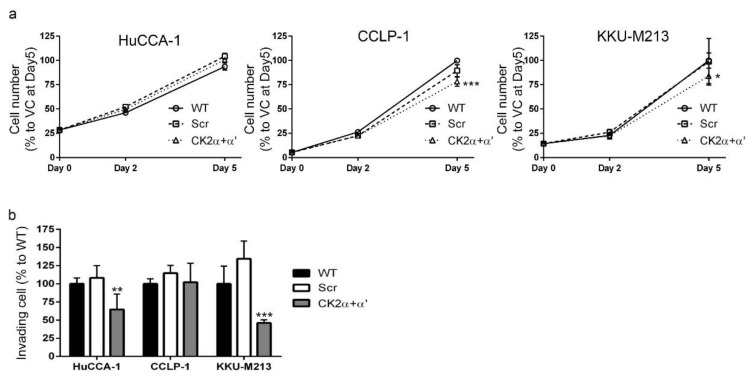
CK2 knockdown had little effect on CCA cell proliferation but inhibited cell invasion. (**a**) Knockdown of α and α′ catalytic subunits of protein kinase CK2 (Figure 7a) decreased CCLP-1 and KKU-M213 cell number as determined using MTT assays. (**b**) Knockdown of α and α′ catalytic subunits of protein kinase CK2 reduced HuCCA-1 and KKU-M213 cell invasion. WT; wild type, Scr; scramble siRNA, *; *p* < 0.05, **; *p* < 0.01, ***; *p* < 0.001. All experiments were performed as at least three biological replicates. Graphs were plotted as mean ± SEM.

To investigate the effects of CK2 α and CK2 α′ knockdown on CCA cell proliferation, we next examined cell number and assayed BrdU incorporation. Combined CK2 α and CK2 α′ knockdown had no effect on HUCCA-1 cell number over 5 days in culture and only a modest effect on the number of CCLP-1 and KKU-M213 cells (Figure 8a). CCLP-1 and KKU-M213 cell number was decreased by 25% at 5 days after gene knockdown. In contrast to the biphasic effects of CX-4945 treatment on CCA cell invasion, combined CK2 α and CK2 α′ knockdown inhibited CCA cell invasion in HuCCA-1 and KKU-M213 cells. HuCCA-1 cell invasion was inhibited to approximately 60% of the control, while KKU-M213 cell invasion was inhibited to approximately 45% of the control (Figure 8b). Moreover, CK2 α and CK2 α′ knockdown had no effect on invasion of CCLP-1 cells. We conclude that the effects of CX-4945 treatment on CCA cell proliferation and cell invasion are unlikely to be mediated by the inhibition of CK2 in these cells.

## 3. Discussion

CX-4945 is in clinical phase I/II trials for cholangiocarcinoma (ClinicalTrials.gov identifier: NCT02128282). Moreover, this drug has shown a therapeutic potential in several other types of cancer [17,22,28,44,45,46]. Herein, CX-4945, at 10 μM and above, was shown to decrease protein kinase CK2 activity when delivered to CCA cells and to inhibit CCA cell proliferation (Figure 1). Unexpectedly, treatment with CX-4945 had biphasic effects on the ability of CCA cells to invade Matrigel. At 10 µM, CX-4945 inhibited CCA cell invasion through a reduction of MMP-9 and MMP-7 levels plus an inhibitory effect on cell proliferation. Interestingly, lower doses of CX-4945 (1 and 5 µM) increased the levels of MMP-9, MMP-7, and MMP-2 (Figure 1e,f). A biphasic effect of CX-4945 on cell proliferation has been reported previously in s22Rv1 prostate cancer cells [25], UM-SCC1 head and neck cancer cells [47], and A549 lung cancer cells [48]. CX-4945 at 0.5, 0.1, and 0.01 µM slightly stimulated cell proliferation in the aforementioned cell lines, whereas higher doses inhibited cell proliferation [25,47,48]. Knockdown of the α and α′ catalytic subunits of CK2 simultaneously also significantly reduced the phosphorylation of multiple CK2 substrates indicating that CK2 knockdown and CX-4945 treatment both inhibited CK2 activity. We measured CK2 activity indirectly by using an antibody against the phospho-CK2 substrate (motif pS/pTDXE) to measure the amount of phosphorylated CK2 substrates. This method is widely used in literature to measure kinase activity, such as activity of the following kinases CK2 [16,49,50,51,52], AMPK [53,54,55,56,57,58,59,60,61,62,63], AMT/ATR [64,65,66,67,68,69,70], PKA [71,72,73,74,75,76,77,78,79], and PKC [77,80,81,82,83,84,85,86,87,88]. Both CK2 knockdown and CX-4945 treatment inhibited the proliferation of CCA cells. However, in contrast to the biphasic effects of CX-4945 on CCA cell invasion, CK2 knockdown only inhibited CCA cell invasion. These data suggest that CX-4945 treatment increases CCA cell invasion independently of CK2 inhibition. In addition, intensive vacuolization and cell death was observed following CX-4945 treatment (Figure 2 and Figure 3), but vacuolization was absent following CK2α and CK2α′ knockdown (Figure 7c) and following treatment with another CK2 inhibitor (Figure 7e–g). This suggests that the induction of vacuole formation is also an off target effect of CX-4945. Recently, an off target effect of CX-4945 was reported. CX-4945 was reported to inhibit the Cdc2-like kinases (Clks) resulting in a suppression of phosphorylation of serine/arginine (S/R) rich proteins in mammalian cells [31]. In addition, CX-4945 has been shown to affect alternative splicing of a wide range of genes in a CK2-independent manner [31]. Inhibition of these or other kinases may be responsible for increased cell invasion and/or increased vacuolization and cell death.

It has been reported that CX-4945 induces apoptosis via caspase-3 activity in lung cancer cells [17] and in primary CCA tumor lines [28]. Autophagy was induced by CX-4945 in pancreatic and lung cancer cell lines [45,89]. Surprisingly, we have shown that CX-4945-induced cell death in CCA cell lines was not accompanied by caspase-3 cleavage and did not appear to involve autophagy (Figure 5a,b) despite the involvement of intensive cytoplasmic vacuolization. This phenomenon was observed in a range of cell lines including CCA cell lines, immortalized cholangiocyte cell lines, breast and prostate cancer cell lines, and HEK293T cells. CX-4945-induced vacuoles originated from an engulfment of extracellular fluid, not from the ER. (Figure 6b,c). Interestingly, non-canonical cell death involving intensive cytoplasmic vacuolization is a hallmark of methuosis, oncosis, paratosis, and necroptosis [35]. We propose that the non-apoptotic, non-autophagic cell death mechanism induced by CX-4945 is not oncosis, paraptosis, or necroptosis according to the following criteria. First, no membrane blebbing was observed (Figure 3a). Second, chromatin condensation and nuclear fragmentation were absent (Figure 6a). Third, the vacuoles originated from an engulfment of extracellular fluid, not from the ER (Figure 6b,c) [35]. Furthermore, the vacuoles merged with others into large vacuoles (Figure 2) and their pH remained neutral (Figure 6c). All of the aforementioned data fit the criteria of methuosis, therefore, we propose that CX-4945 induces methuosis at least in the CCA cell line KKU-M213.

Interestingly, PC3 prostate cancer cells did not form vacuoles. This absence of the vacuoles in PC3 cells suggests a cell line specific response to CX-4945. It seems likely that CX-4945 activity and its effects on cell death are cell-type specific, that is, dependent on, for example, whether tumor suppressors such as TP53 are wild type or mutant in the cell line being tested. Further study on the PC3 cell line might elucidate the molecular mechanism underlying the CX-4945-induced vacuolization and cell death reported in this study. Further investigation of the molecular mechanisms underlying CX-4945-induced vacuolization and cell death might lead to a better understanding of possible combinations of this treatment with other current chemotherapeutic drugs to optimize treatment outcomes in patients.

## 4. Materials and Methods

### 4.1. Cell Culture

Two cholangiocarcinoma cell lines, KKU-M055 and KKU-M213, established as previously described [90], were purchased from the Japanese Collection of Research Bioresources (JCRB) Cell Bank (Osaka, Japan). These two cell lines were from a Thai patient with an *Opisthorchis viverrini* record. The immortalized cholangiocytes cell line, MMNK-1, was also purchased from the JCRB Cell Bank. Intraheptic cholangiocarcinoma cell lines, HuCCA-1 were established and kindly provided by Prof. Stitaya Sirisinha [91]. HuCCA-1 was derived from a Thai patient whose serum was positive for the *O. viverrini* antigen [91]. Prostate cancer cell lines, PC3 and DU145, were purchased from American Type Culture Collection (ATCC) (Manassas, VA, USA). The immortalized human cholangiocytes cell line, AKN-1, and cholangiocarcinoma cell lines, CCLP-1, were kindly provided by Dr. Simon Afford. Breast cancer cell lines, MDA-MB-231, T47D, MCF7, and HEK293T, were purchased from ATCC (Manassas, VA, USA). HuCCA-1 and PC3 cells were maintained in HAM’s F-12 medium (Hyclone, Pittsburgh, PA, USA) supplemented with 10% fetal bovine serum (FBS) (Gibco, Grand Island, NY, USA), while CCLP-1, KKU-M055, KKU-M213, MMNK-1, AKN-1, DU145, HEK293T, MDA-MB-231, T47D, and MCF7 cells were maintained in Dulbecco’s modified Eagle’s medium (DMEM) (Hyclone, Pittsburgh, PA, USA) supplemented with 10% FBS and 1% MEM non-essential amino acid (Gibco, Grand Island, NY, USA). All media were supplemented with 1% Penicillin/Streptomycin (Gibco, Grand Island, NY, USA). All cell lines were maintained at 37 °C with 5% CO_2_.

### 4.2. MTT Assay

Cells were plated in tissue culture treated 96-well plates. After overnight adhesion to the plate, the treatment group was incubated with the indicated concentrations of CX-4945 (Santa Cruz Biotechnology, Dallas, TX, USA) or 4,5,6,7-tetrabromo-2-azabenzimidazole, 4,5,6,7-Tetrabromobenzotriazole (TBB) (Sigma Aldrich, St. Louis, MO, USA). Media were changed and new treatments were added on day 2. On day 0, 2, and 5, MTT reagent (Fisher Scientific, Hampton, NH, USA) was added to the final concentration of 0.5 mg/mL and incubated for 2.5 h at 37 °C. A half volume of stop solution (10% Sodium dodecyl sulfate (SDS) in 50% dimethylformamide in dH_2_O) was added and mixed thoroughly before reading the absorbance at 560 nm on a plate reader.

### 4.3. BrdU Incorporation Assay

Cells were plated in tissue culture treated 96-well plates. BrdU incorporation was assessed by using a BrdU Cell Proliferation kit (ab126556, Abcam, Cambridge, MA, USA). Cells were treated with CX-4945 for 24 h. During the last 6 h, cells were incubated in a working stock of BrdU. After incubation with BrdU, media was removed and a fixative was added and the plates were incubated for 30 min at 23 °C. Reaction cocktail was added according to the manufacturer’s protocol. The plate was read with a spectrophotometer plate reader at an absorbance at 450 nm. The average no BrdU background measurement was subtracted from all experimental measurements.

### 4.4. Cell Invasion Assay

Eight micrometer polycarbonate membrane transwell inserts (Corning costar, New York, NY, USA) were coated with 50 µL of a 1:9 ratio of Matrigel™ (BD Biosciences, San Jose, CA, USA) to serum free medium, that was allowed to congeal at 37 °C for 30 min. A total of 5 × 10^4^ cells was added to the top chamber of each well in serum free medium. The bottom well was filled with complete medium. Treatment was added in both the transwell and the bottom well. Cells were incubated for 24 h at a 37 °C in a humidified incubator with 5% CO_2_. The chambers were swabbed to remove cells and the Matrigel™ remaining on the top of the membrane. Cells on the underside of the membrane were fixed in methanol for 5 min and stained with 0.5% (*w*/*v*) crystal violet in 12% glutaraldehyde in water for 5 min. Following a brief distilled H_2_O wash, cells were counted using a Nikon Eclipse T2S phase contrast inverted fluorescent microscope.

### 4.5. Cell Viability Assay

Cells were plated in tissue culture treated 6-well plates and incubated overnight. CX-4945 treatments were added and incubated for either 4 h or 48 h. Calcein-acetoxymethyl (AM) (Fisher Scientific) and propidium iodide (Fisher Scientific) were added 30 min before imaging at a final concentration of 0.125 µM and 1.5 µM, respectively. Images were taken with Nikon Eclipse T2S phase contrast inverted fluorescent microscope. Live cells were stained with calcein and showed a green fluorescent signal while dead cells were stained with Propidium iodine and showed a red fluorescent signal. Green and red fluorescence pixels were counted using Adobe Photoshop software. Average live and dead cells sizes were measured using Adobe Photoshop software.

### 4.6. Western Blot Analysis

A total of 20 µg of protein was separated electrophoretically and transferred to nitrocellulose membrane (GE, Boston, MA, USA) at room temperature. Membranes were blocked in a blocking buffer (4% bovine serum albumin (BSA) *w*/*v* in Tris-buffered saline, 0.1% Tween 20 (TBST)), and then incubated with primary antibodies overnight at 4 °C on a rocking shaker. All antibodies were diluted in blocking buffer. All primary antibodies were monoclonal rabbit anti-human antibodies. Primary antibodies included 1:1500 MMP-9 (2270, Cell Signaling Technology, Danvers, MA, USA), 1:1000 MMP-7 (ab5706, Abcam), 1:1500 MMP-2 (4022, Cell Signaling Technology), 1:1500 cleaved caspase-3 (9664, Cell Signaling Technology), 1:1500 phospho-CK2 substrate (motif pS/pTDXE) (8738, Cell Signaling Technology), 1:1500 LC3B (3868, Cell Signaling Technology), and 1:5000 β-actin (A2066, Sigma Aldrich, St. Louis, MO, USA). Membranes were incubated for 75 min on a rocking shaker at 23 °C with secondary antibodies. Secondary antibodies included 1:5000 goat anti-rabbit IgG conjugated with horseradish peroxidase (7074, Cell Signaling Technology) or 1:5000 horse anti-mouse immunoglobulin G (IgG) conjugated with horseradish peroxidase (7076, Cell Signaling Technology). Signal was visualized by autoradiography using enhanced chemiluminescence (ECL) and a horseradish peroxidase (HRP) chemiluminescent substrate (Millipore, Burlington, MA, USA), and exposed to Hyperfilm (GE Healthcare, Boston, MA, USA). Bands intensity was analyzed using ImageJ software.

### 4.7. siRNA Transfection

Transfection was performed by adding 4 × 10^5^ cells to 100 nM siRNA mixture in Optimem medium (Fisher Scientific) in a total volume of 500 µL. siRNAs were commercially available from Qaigen (CK2α SI02660504 and SI02660497, CK2α′ SI00605416 and SI00605409). The mixture of siRNA and cells was plated into 6-well plates and incubated in a 37 °C humidified incubator with 5% CO_2_ for 4 h. Complete growth medium was then added and incubated for 2 days before cells were used in further experiments.

### 4.8. Organelle Labeling and Imaging

Cells were plated in tissue culture treated 6-well plates and incubated overnight. CellLight™ ER-GFP and BacMam 2.0 (C10590, Fisher Scientific) were added to cells and incubated for 72 h at 50 particles per cell. LysoSensor™ Yellow/Blue DND-160 (L7545 Fisher Scientific) was added 15 min before the treatment. Treatment (25 µM CX-4945) was added and incubated for 6 h. Fresh culture media was changed prior to imaging. Live cells were imaged with a fluorescent invested microscope with cell culture chamber module (Olympus) at the Olympus Imaging Center, Mahidol University. Cells were plated on circle cover slides in tissue culture 24-well plates and incubated overnight. Twenty-five micromolars of CX-4945 was added and incubated for 24 h. Cells were fixed in 3.7% formaldehyde in Phosphate buffered saline (PBS) for 10 min, and then permeabilized in 0.1% Triton X-100 in PBS for 2 min. After incubation, cells were incubated in 300 nM DAPI in PBS to stain the nuclei. Fixed cells were imaged with a confocal microscope (Olympus) at the Olympus Imaging Center, Mahidol University.

### 4.9. Statistical Analysis

Data were graphed as mean ± standard error of the mean (SEM) using Prism Graphpad software (version 6.01, GraphPad Software, La Jolla, CA, USA). Statistical analyses were performed using ANOVA with Dunnett’s test by using JMP software (version 10.0.0, SAS Institute Inc., Cary, NC, USA). All experiments were performed in triplicate.

## 5. Conclusions

Our data suggest that CX-4945 inhibits cell proliferation and induces methuosis via CK2-independent pathways. A biphasic effect of CX-4945 on CCA cell invasion was, at least in part, due to an alteration of MMP-2, MMP-7, and MMP-9.

## References

[1] Njei B. (2014). Changing pattern of epidemiology in intrahepatic cholangiocarcinoma. Hepatology.

[2] Pinter M., Hucke F., Zielonke N., Waldhor T., Trauner M., Peck-Radosavljevic M., Sieghart W. (2014). Incidence and mortality trends for biliary tract cancers in austria. Liver Int..

[3] Tanaka A., Takikawa H. (2013). Geoepidemiology of primary sclerosing cholangitis: A critical review. J. Autoimmun..

[4] Gupta A., Dixon E. (2017). Epidemiology and risk factors: Intrahepatic cholangiocarcinoma. Hepatobiliary Surg. Nutr..

[5] Furst T., Keiser J., Utzinger J. (2012). Global burden of human food-borne trematodiasis: A systematic review and meta-analysis. Lancet Infect. Dis..

[6] Kaewpitoon S.J., Rujirakul R., Ueng-Arporn N., Matrakool L., Namwichaisiriku N., Churproong S., Wongkaewpothong P., Nimkuntod P., Sripa B., Kaewpitoon N. (2012). Community-based cross-sectional study of carcinogenic human liver fluke in elderly from Surin province, Thailand. Asian Pac. J. Cancer Prev..

[7] Sithithaworn P., Yongvanit P., Duenngai K., Kiatsopit N., Pairojkul C. (2014). Roles of liver fluke infection as risk factor for cholangiocarcinoma. J. Hepatobiliary Pancreat. Sci..

[8] Bridgewater J., Galle P.R., Khan S.A., Llovet J.M., Park J.W., Patel T., Pawlik T.M., Gores G.J. (2014). Guidelines for the diagnosis and management of intrahepatic cholangiocarcinoma. J. Hepatol..

[9] Yoh T., Hatano E., Yamanaka K., Nishio T., Seo S., Taura K., Yasuchika K., Okajima H., Kaido T., Uemoto S. (2016). Is surgical resection justified for advanced intrahepatic cholangiocarcinoma?. Liver Cancer.

[10] Zhang X.F., Beal E.W., Bagante F., Chakedis J., Weiss M., Popescu I., Marques H.P., Aldrighetti L., Maithel S.K., Pulitano C. (2017). Early versus late recurrence of intrahepatic cholangiocarcinoma after resection with curative intent. Br. J. Surg..

[11] Primrose J.N., Fox R., Palmer D.H., Prasad R., Mirza D., Anthoney D.A., Corrie P., Falk S., Wasan H.S., Ross P.J. (2017). Adjuvant capecitabine for biliary tract cancer: The bilcap randomized study. J. Clin. Oncol..

[12] Valle J., Wasan H., Palmer D.H., Cunningham D., Anthoney A., Maraveyas A., Madhusudan S., Iveson T., Hughes S., Pereira S.P. (2010). Cisplatin plus gemcitabine versus gemcitabine for biliary tract cancer. N. Engl. J. Med..

[13] Litchfield D.W. (2003). Protein kinase ck2: Structure, regulation and role in cellular decisions of life and death. Biochem. J.

[14] Ortega C.E., Seidner Y., Dominguez I. (2014). Mining CK2 in cancer. PLoS ONE.

[15] Wang J., Feng C., He Y., Ding W., Sheng J., Arshad M., Zhang X., Li P. (2015). Phosphorylation of apoptosis repressor with caspase recruitment domain by protein kinase CK2 contributes to chemotherapy resistance by inhibiting doxorubicin induced apoptosis. Oncotarget.

[16] Kendall J.J., Chaney K.E., Patel A.V., Rizvi T.A., Largaespada D.A., Ratner N. (2016). CK2 blockade causes mpnst cell apoptosis and promotes degradation of beta-catenin. Oncotarget.

[17] So K.S., Rho J.K., Choi Y.J., Kim S.Y., Choi C.M., Chun Y.J., Lee J.C. (2015). AKT/mTOR down-regulation by CX-4945, a CK2 inhibitor, promotes apoptosis in chemorefractory non-small cell lung cancer cells. Anticancer Res..

[18] Quotti Tubi L., Gurrieri C., Brancalion A., Bonaldi L., Bertorelle R., Manni S., Pavan L., Lessi F., Zambello R., Trentin L. (2013). Inhibition of protein kinase CK2 with the clinical-grade small ATP-competitive compound CX-4945 or by rna interference unveils its role in acute myeloid leukemia cell survival, p53-dependent apoptosis and daunorubicin-induced cytotoxicity. J. Hematol. Oncol..

[19] Zhang F., Yang B., Shi S., Jiang X. (2014). RNA interference (RNAi) mediated stable knockdown of protein casein kinase 2-alpha (CK2alpha) inhibits migration and invasion and enhances cisplatin-induced apoptosis in HEp-2 laryngeal carcinoma cells. Acta Histochem..

[20] Ryu S.-W., Woo J.H., Kim Y.-H., Lee Y.-S., Park J.W., Bae Y.-S. (2006). Downregulation of protein kinase CKII is associated with cellular senescence. FEBS Lett..

[21] Yao K., Youn H., Gao X., Huang B., Zhou F., Li B., Han H. (2012). Casein kinase 2 inhibition attenuates androgen receptor function and cell proliferation in prostate cancer cells. Prostate.

[22] Zhang X., Yang X., Yang C., Li P., Yuan W., Deng X., Cheng Y., Li P., Yang H., Tao J. (2016). Targeting protein kinase CK2 suppresses bladder cancer cell survival via the glucose metabolic pathway. Oncotarget.

[23] Gowda C., Sachdev M., Muthusami S., Kapadia M., Petrovic-Dovat L., Hartman M., Ding Y., Song C., Payne J.L., Tan B.H. (2017). Casein kinase II (CK2) as a therapeutic target for hematological malignancies. Curr. Pharm. Des..

[24] Lian H., Li D., Zhou Y., Landesman-Bollag E., Zhang G., Anderson N.M., Tang K.C., Roderick J.E., Kelliher M.A., Seldin D.C. (2017). CK2 inhibitor CX-4945 destabilizes NOTCH1 and synergizes with JQ1 against human T-acute lymphoblastic leukemic cells. Haematologica.

[25] Deng C., Chen J., Guo S., Wang Y., Zhou Q., Li Z., Yang X., Yu X., Zhang Z., Zhou F. (2017). CX4945 suppresses the growth of castration-resistant prostate cancer cells by reducing AR-V7 expression. World J. Urol..

[26] Gray G.K., McFarland B.C., Rowse A.L., Gibson S.A., Benveniste E.N. (2014). Therapeutic CK2 inhibition attenuates diverse prosurvival signaling cascades and decreases cell viability in human breast cancer cells. Oncotarget.

[27] Pierre F., Chua P.C., O’Brien S.E., Siddiqui-Jain A., Bourbon P., Haddach M., Michaux J., Nagasawa J., Schwaebe M.K., Stefan E. (2011). Pre-clinical characterization of CX-4945, a potent and selective small molecule inhibitor of CK2 for the treatment of cancer. Mol. Cell. Biochem..

[28] Lustri A.M., Di Matteo S., Fraveto A., Costantini D., Cantafora A., Napoletano C., Bragazzi M.C., Giuliante F., De Rose A.M., Berloco P.B. (2017). TGF-beta signaling is an effective target to impair survival and induce apoptosis of human cholangiocarcinoma cells: A study on human primary cell cultures. PLoS ONE.

[29] Olsen B.B., Issinger O.G., Guerra B. (2010). Regulation of DNA-dependent protein kinase by protein kinase CK2 in human glioblastoma cells. Oncogene.

[30] Willmore E., de Caux S., Sunter N.J., Tilby M.J., Jackson G.H., Austin C.A., Durkacz B.W. (2004). A novel DNA-dependent protein kinase inhibitor, NU7026, potentiates the cytotoxicity of topoisomerase II poisons used in the treatment of leukemia. Blood.

[31] Kim H., Choi K., Kang H., Lee S.Y., Chi S.W., Lee M.S., Song J., Im D., Choi Y., Cho S. (2014). Identification of a novel function of CX-4945 as a splicing regulator. PLoS ONE.

[32] Galluzzi L., Vitale I., Abrams J.M., Alnemri E.S., Baehrecke E.H., Blagosklonny M.V., Dawson T.M., Dawson V.L., El-Deiry W.S., Fulda S. (2012). Molecular definitions of cell death subroutines: Recommendations of the nomenclature committee on cell death 2012. Cell Death Differ..

[33] Lockshin R.A., Zakeri Z. (2004). Apoptosis, autophagy, and more. Int. J. Biochem. Cell Biol..

[34] Kroemer G., Levine B. (2008). Autophagic cell death: The story of a misnomer. Nat. Rev. Mol. Cell Biol..

[35] Maltese W.A., Overmeyer J.H. (2014). Methuosis: Nonapoptotic cell death associated with vacuolization of macropinosome and endosome compartments. Am. J. Pathol..

[36] Overmeyer J.H., Kaul A., Johnson E.E., Maltese W.A. (2008). Active ras triggers death in glioblastoma cells through hyperstimulation of macropinocytosis. Mol. Cancer Res..

[37] Overmeyer J.H., Young A.M., Bhanot H., Maltese W.A. (2011). A chalcone-related small molecule that induces methuosis, a novel form of non-apoptotic cell death, in glioblastoma cells. Mol. Cancer.

[38] Weerasinghe P., Buja L.M. (2012). Oncosis: An important non-apoptotic mode of cell death. Exp. Mol. Pathol..

[39] Hitomi J., Christofferson D.E., Ng A., Yao J., Degterev A., Xavier R.J., Yuan J. (2008). Identification of a molecular signaling network that regulates a cellular necrotic cell death pathway. Cell.

[40] Degterev A., Huang Z., Boyce M., Li Y., Jagtap P., Mizushima N., Cuny G.D., Mitchison T.J., Moskowitz M.A., Yuan J. (2005). Chemical inhibitor of nonapoptotic cell death with therapeutic potential for ischemic brain injury. Nat. Chem. Biol..

[41] Sperandio S., de Belle I., Bredesen D.E. (2000). An alternative, nonapoptotic form of programmed cell death. Proc. Natl. Acad. Sci. USA.

[42] Majno G., Joris I. (1995). Apoptosis, oncosis, and necrosis. An overview of cell death. Am. J. Pathol..

[43] Li Z., Mbah N.E., Maltese W.A. (2018). Vacuole-inducing compounds that disrupt endolysosomal trafficking stimulate production of exosomes by glioblastoma cells. Mol. Cell. Biochem..

[44] Chon H.J., Bae K.J., Lee Y., Kim J. (2015). The casein kinase 2 inhibitor, CX-4945, as an anti-cancer drug in treatment of human hematological malignancies. Front. Pharmacol..

[45] Hwang D.W., So K.S., Kim S.C., Park K.M., Lee Y.J., Kim S.W., Choi C.M., Rho J.K., Choi Y.J., Lee J.C. (2017). Autophagy induced by CX-4945, a casein kinase 2 inhibitor, enhances apoptosis in pancreatic cancer cell lines. Pancreas.

[46] Quotti Tubi L., Canovas Nunes S., Brancalion A., Doriguzzi Breatta E., Manni S., Mandato E., Zaffino F., Macaccaro P., Carrino M., Gianesin K. (2017). Protein kinase CK2 regulates AKT, NF-kappaB and STAT3 activation, stem cell viability and proliferation in acute myeloid leukemia. Leukemia.

[47] Bian Y., Han J., Kannabiran V., Mohan S., Cheng H., Friedman J., Zhang L., VanWaes C., Chen Z. (2015). MEK inhibitor PD-0325901 overcomes resistance to CK2 inhibitor CX-4945 and exhibits anti-tumor activity in head and neck cancer. Int. J. Biol. Sci..

[48] Zhang S., Long H., Yang Y.L., Wang Y., Hsieh D., Li W., Au A., Stoppler H.J., Xu Z., Jablons D.M. (2013). Inhibition of CK2alpha down-regulates Notch1 signalling in lung cancer cells. J. Cell. Mol. Med..

[49] Wadey K.S., Brown B.A., Sala-Newby G.B., Jayaraman P.S., Gaston K., George S.J. (2017). Protein kinase CK2 inhibition suppresses neointima formation via a proline-rich homeodomain-dependent mechanism. Vascul. Pharmacol..

[50] von Morgen P., Burdova K., Flower T.G., O’Reilly N.J., Boulton S.J., Smerdon S.J., Macurek L., Horejsi Z. (2017). MRE11 stability is regulated by CK2-dependent interaction with R2TP complex. Oncogene.

[51] Shu S., Lin C.Y., He H.H., Witwicki R.M., Tabassum D.P., Roberts J.M., Janiszewska M., Huh S.J., Liang Y., Ryan J. (2016). Response and resistance to BET bromodomain inhibitors in triple-negative breast cancer. Nature.

[52] Greene T.T., Tokuyama M., Knudsen G.M., Kunz M., Lin J., Greninger A.L., DeFilippis V.R., DeRisi J.L., Raulet D.H., Coscoy L. (2016). A herpesviral induction of RAE-1 NKG2D ligand expression occurs through release of HDAC mediated repression. eLife.

[53] Li F.L., Liu J.P., Bao R.X., Yan G., Feng X., Xu Y.P., Sun Y.P., Yan W., Ling Z.Q., Xiong Y. (2018). Acetylation accumulates PFKFB3 in cytoplasm to promote glycolysis and protects cells from cisplatin-induced apoptosis. Nat. Commun..

[54] Zhu Q., Ghoshal S., Tyagi R., Chakraborty A. (2017). Global IP6K1 deletion enhances temperature modulated energy expenditure which reduces carbohydrate and fat induced weight gain. Mol. Metab..

[55] Calamita P., Miluzio A., Russo A., Pesce E., Ricciardi S., Khanim F., Cheroni C., Alfieri R., Mancino M., Gorrini C. (2017). SBDS-deficient cells have an altered homeostatic equilibrium due to translational inefficiency which explains their reduced fitness and provides a logical framework for intervention. PLoS Genet..

[56] Zhang D., Wang W., Sun X., Xu D., Wang C., Zhang Q., Wang H., Luo W., Chen Y., Chen H. (2016). AMPK regulates autophagy by phosphorylating BECN1 at threonine 388. Autophagy.

[57] Johanns M., Lai Y.C., Hsu M.F., Jacobs R., Vertommen D., Van Sande J., Dumont J.E., Woods A., Carling D., Hue L. (2016). AMPK antagonizes hepatic glucagon-stimulated cyclic AMP signalling via phosphorylation-induced activation of cyclic nucleotide phosphodiesterase 4B. Nat. Commun..

[58] Di Magno L., Basile A., Coni S., Manni S., Sdruscia G., D’Amico D., Antonucci L., Infante P., De Smaele E., Cucchi D. (2016). The energy sensor AMPK regulates hedgehog signaling in human cells through a unique Gli1 metabolic checkpoint. Oncotarget.

[59] Fu X., Zhu M.J., Dodson M.V., Du M. (2015). AMP-activated protein kinase stimulates warburg-like glycolysis and activation of satellite cells during muscle regeneration. J. Biol. Chem..

[60] Lu K., Wang L., Wang C., Yang Y., Hu D., Ding R. (2015). Effects of high-intensity interval versus continuous moderate-intensity aerobic exercise on apoptosis, oxidative stress and metabolism of the infarcted myocardium in a rat model. Mol. Med. Rep..

[61] Obba S., Hizir Z., Boyer L., Selimoglu-Buet D., Pfeifer A., Michel G., Hamouda M.A., Goncalves D., Cerezo M., Marchetti S. (2015). The PRKAA1/AMPkalpha1 pathway triggers autophagy during CSF1-induced human monocyte differentiation and is a potential target in CMML. Autophagy.

[62] Ducommun S., Deak M., Sumpton D., Ford R.J., Nunez Galindo A., Kussmann M., Viollet B., Steinberg G.R., Foretz M., Dayon L. (2015). Motif affinity and mass spectrometry proteomic approach for the discovery of cellular AMPK targets: Identification of mitochondrial fission factor as a new AMPK substrate. Cell. Signal..

[63] Choi D.W., Na W., Kabir M.H., Yi E., Kwon S., Yeom J., Ahn J.W., Choi H.H., Lee Y., Seo K.W. (2013). WIP1, a homeostatic regulator of the DNA damage response, is targeted by HIPK2 for phosphorylation and degradation. Mol. Cell.

[64] Zhang W., Huang Y., Gunst S.J. (2016). P21-activated kinase (Pak) regulates airway smooth muscle contraction by regulating paxillin complexes that mediate actin polymerization. J. Physiol..

[65] Ahlskog J.K., Larsen B.D., Achanta K., Sorensen C.S. (2016). ATM/ATR-mediated phosphorylation of PALB2 promotes RAD51 function. EMBO Rep..

[66] Hao J., de Renty C., Li Y., Xiao H., Kemp M.G., Han Z., DePamphilis M.L., Zhu W. (2015). And-1 coordinates with claspin for efficient Chk1 activation in response to replication stress. EMBO J..

[67] Rath A., Hromas R., De Benedetti A. (2014). Fidelity of end joining in mammalian episomes and the impact of Metnase on joint processing. BMC Mol. Biol..

[68] Hori T., Uemoto S., Chen F., Gardner L.B., Baine A.M., Hata T., Kogure T., Nguyen J.H. (2014). Oxidative stress and extracellular matrices after hepatectomy and liver transplantation in rats. World J. Hepatol..

[69] Hori T., Gardner L.B., Hata T., Chen F., Baine A.M., Uemoto S., Nguyen J.H. (2013). Pretreatment of liver grafts in vivo by gamma-aminobutyric acid receptor regulation reduces cold ischemia/warm reperfusion injury in rat. Ann. Transpl..

[70] Gardner L.B., Hori T., Chen F., Baine A.M., Hata T., Uemoto S., Nguyen J.H. (2012). Effect of specific activation of gamma-aminobutyric acid receptor in vivo on oxidative stress-induced damage after extended hepatectomy. Hepatol. Res..

[71] Ando F., Mori S., Yui N., Morimoto T., Nomura N., Sohara E., Rai T., Sasaki S., Kondo Y., Kagechika H. (2018). AKAPs-PKA disruptors increase AQP2 activity independently of vasopressin in a model of nephrogenic diabetes insipidus. Nat. Commun..

[72] Liu D., Ceddia R.P., Collins S. (2018). Cardiac natriuretic peptides promote adipose ‘browning’ through mTOR complex-1. Mol. Metab..

[73] Agarwal S., Cho T.Y. (2018). Biochemical and structural characterization of a novel cooperative binding mode by Pit-1 with CATT repeats in the macrophage migration inhibitory factor promoter. Nucleic Acids Res..

[74] Taylor E.J.A., Pantazaka E., Shelley K.L., Taylor C.W. (2017). Prostaglandin E_2_ inhibits histamine-evoked Ca^2+^ release in human aortic smooth muscle cells through hyperactive cAMP signaling junctions and protein kinase A. Mol. Pharmacol..

[75] Mohl B.P., Emmott E., Roy P. (2017). Phosphoproteomic analysis reveals the importance of kinase regulation during orbivirus infection. Mol. Cell. Proteom..

[76] Jensen P., Myhre C.L., Lassen P.S., Metaxas A., Khan A.M., Lambertsen K.L., Babcock A.A., Finsen B., Larsen M.R., Kempf S.J. (2017). TNFalpha affects CREB-mediated neuroprotective signaling pathways of synaptic plasticity in neurons as revealed by proteomics and phospho-proteomics. Oncotarget.

[77] Okamoto Y., Shikano S. (2017). Differential phosphorylation signals control endocytosis of GPR15. Mol. Biol. Cell.

[78] Garcia R., Bravo E., Diez-Muniz S., Nombela C., Rodriguez-Pena J.M., Arroyo J. (2017). A novel connection between the cell wall integrity and the PKA pathways regulates cell wall stress response in yeast. Sci. Rep..

[79] Hiday A.C., Edler M.C., Salek A.B., Morris C.W., Thang M., Rentz T.J., Rose K.L., Jones L.M., Baucum A.J. (2017). Mechanisms and consequences of dopamine depletion-induced attenuation of the spinophilin/neurofilament medium interaction. Neural Plast..

[80] Shyu K.G., Velusamy M., Hsia C.W., Yang C.H., Hsia C.H., Chou D.S., Jayakumar T., Sheu J.R., Li J.Y. (2018). Novel iridium (III)derived organometallic compound for the inhibition of human platelet activation. Int. J. Mol. Med..

[81] Monteverde T., Tait-Mulder J., Hedley A., Knight J.R., Sansom O.J., Murphy D.J. (2018). Calcium signalling links MYC to NUAK1. Oncogene.

[82] Zitouni S., Mechali F., Papin C., Choquet A., Roche D., Baldin V., Coux O., Bonne-Andrea C. (2017). The stability of Fbw7alpha in M-phase requires its phosphorylation by PKC. PLoS ONE.

[83] Waschek J.A., Cohen J.R., Chi G.C., Proszynski T.J., Niewiadomski P. (2017). PACAP promotes matrix-driven adhesion of cultured adult murine neural progenitors. ASN Neuro.

[84] Tello-Lafoz M., Rodriguez-Rodriguez C., Kinna G., Loo L.S., Hong W., Collins B.M., Teasdale R.D., Merida I. (2017). SNX27 links DGKzeta to the control of transcriptional and metabolic programs in T lymphocytes. Sci. Rep..

[85] Shirafuji T., Ueyama T., Tanaka S., Hide I., Saito N., Sakai N. (2017). Validation of anti-cspalpha, snap25, tyrosine hydroxylase, ubiquitin, cleaved caspase 3, and pser pkc motif antibodies for utilization in western blotting. Acta Histochem. Cytochem..

[86] Lucien F., Pelletier P.P., Lavoie R.R., Lacroix J.M., Roy S., Parent J.L., Arsenault D., Harper K., Dubois C.M. (2017). Hypoxia-induced mobilization of NHE6 to the plasma membrane triggers endosome hyperacidification and chemoresistance. Nat. Commun..

[87] Li P., Stumpf M., Muller R., Eichinger L., Glockner G., Noegel A.A. (2017). The function of the inner nuclear envelope protein SUN1 in mRNA export is regulated by phosphorylation. Sci. Rep..

[88] Carmon K.S., Gong X., Yi J., Wu L., Thomas A., Moore C.M., Masuho I., Timson D.J., Martemyanov K.A., Liu Q.J. (2017). LGR5 receptor promotes cell-cell adhesion in stem cells and colon cancer cells via the IQGAP1-Rac1 pathway. J. Biol. Chem..

[89] So K.S., Kim C.H., Rho J.K., Kim S.Y., Choi Y.J., Song J.S., Kim W.S., Choi C.M., Chun Y.J., Lee J.C. (2014). Autophagosome-mediated EGFR down-regulation induced by the CK2 inhibitor enhances the efficacy of EGFR-TKI on EGFR-mutant lung cancer cells with resistance by T790M. PLoS ONE.

[90] Sripa B., Leungwattanawanit S., Nitta T., Wongkham C., Bhudhisawasdi V., Puapairoj A., Sripa C., Miwa M. (2005). Establishment and characterization of an opisthorchiasis-associated cholangiocarcinoma cell line (KKU-100). World J. Gastroenterol..

[91] Sirisinha S., Tengchaisri T., Boonpucknavig S., Prempracha N., Ratanarapee S., Pausawasdi A. (1991). Establishment and characterization of a cholangiocarcinoma cell line from a thai patient with intrahepatic bile duct cancer. Asian Pac. J. Allergy Immunol..

