# Correction: Lertsuwan et al. CX-4945 Induces Methuosis in Cholangiocarcinoma Cell Lines by a CK2-Independent Mechanism. *Cancers* 2018, *10*, 283

**DOI:** 10.3390/cancers15010133

**Published:** 2022-12-26

**Authors:** Jomnarong Lertsuwan, Kornkamon Lertsuwan, Anyaporn Sawasdichai, Nathapol Tasnawijitwong, Ka Ying Lee, Philip Kitchen, Simon Afford, Kevin Gaston, Padma-Sheela Jayaraman, Jutamaad Satayavivad

**Affiliations:** 1 Laboratory of Chemical Carcinogenesis, Chulabhorn Research Institute, Bangkok 10210, Thailand; 2 Department of Biochemistry, Faculty of Science, Mahidol University, Rama VI Road, Bangkok 10400, Thailand; 3 Center of Calcium and Bone Research (COCAB), Faculty of Science, Mahidol University, Rama VI Road, Bangkok 10400, Thailand; 4 Laboratory of Pharmacology, Chulabhorn Research Institute, Bangkok 10210, Thailand; 5 Institute of Cancer and Genomic Sciences, University of Birmingham, Birmingham B15 2TT, UK; 6 Institute of Immunology and Immunotherapy, University of Birmingham, Birmingham B15 2TT, UK; 7 Division of Cancer and Stem Cells, School of Medicine, University of Nottingham, Nottingham NG7 2RD, UK

Authors would like to make a correction to the previous article [1]. In Figure 1a, Figure 6a, and Figure 7b,d, errors were introduced in the preparation of these figures for publication. 

The errors included:

(1) Figure 1a. Mislabeling the size of β-actin to 40 kDa. The correct size should be 42 kDa.
(2) Figure 6a. Picture of KKU-213 cells in the control group (DIC+DAPI) was mistakenly replaced by HuCCA-1 cells. However, the picture of KKU-213 in the control group that shows only DAPI was correct.
(3) Figure 7b,d. Mislabeling the size of β-actin to 40 kDa. The correct size should be 42 kDa.

The authors have provided the corrected versions of all figures as below:

Figure 1

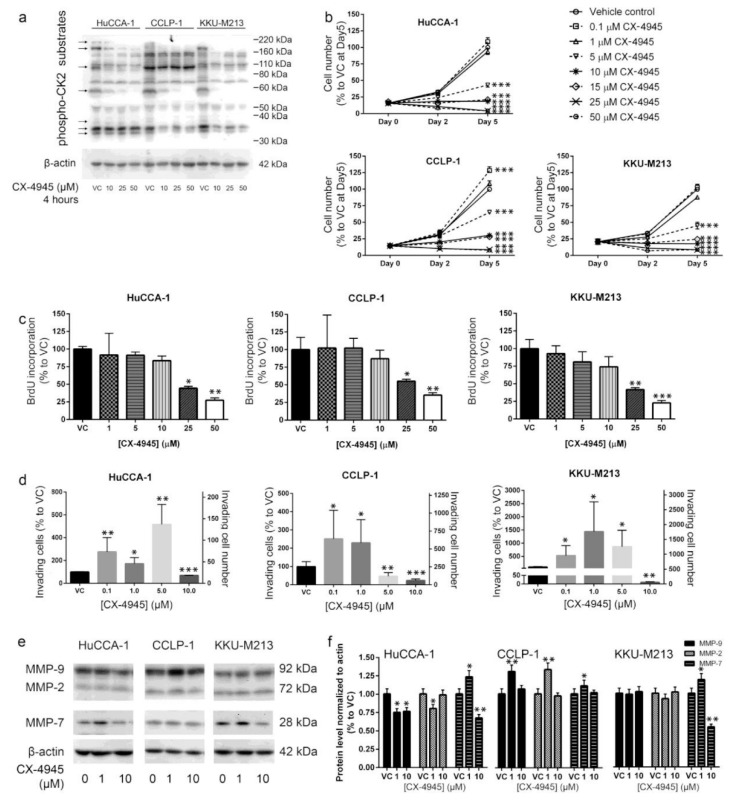



Figure 6

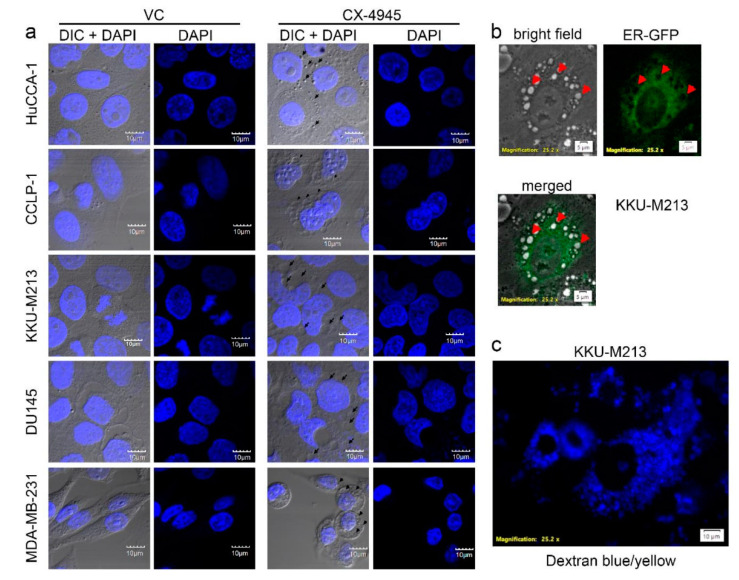



Figure 7

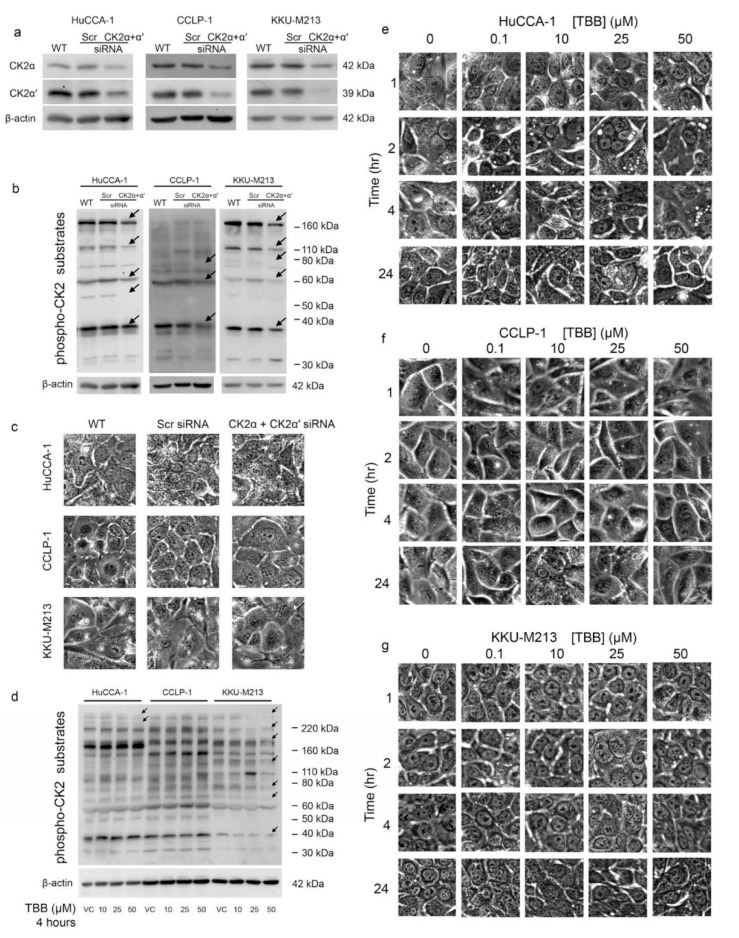



The authors apologize for any inconvenience caused and state that the scientific conclusions are unaffected. The original publication has also been updated.

## References

[1] Lertsuwan J., Lertsuwan K., Sawasdichai A., Tasnawijitwong N., Lee K.Y., Kitchen P., Afford S., Gaston K., Jayaraman P.-S., Satayavivad J. (2018). CX-4945 Induces Methuosis in Cholangiocarcinoma Cell Lines by a CK2-Independent Mechanism. Cancers.

